# Draft Genome Sequence of *Kurthia* Strain ISK08 Isolated from Shrimp in Bangladesh

**DOI:** 10.1128/mra.01337-24

**Published:** 2025-03-25

**Authors:** Md. Imran Khan Masum, Margia Hossain Rahi, Raidah Jahan, Farishta Shahel, Hamja Hasanat, Jinath Sultana Jime, Nayeema Bulbul, Md. Fakruddin, Md. Mainul Hossain, Maqsud Hossain, Abdus Sadique, Ashrafus Safa

**Affiliations:** 1 Department of Life Sciences (DLS), School of Environment and Life Sciences (SELS), Independent University, Bangladesh (IUB) 421815, Dhaka, Bangladesh; 2 Department of Biochemistry and Microbiology, School of Health and Life Sciences, North South University 54495, Dhaka, Bangladesh; Montana State University, Bozeman, Montana, USA

**Keywords:** *Kurthia*, genome analysis, shrimp

## Abstract

We announce the draft genome sequence of the *Kurthia* ISK08 strain, isolated from the shrimp sample. The genome sequence is 2,885,242 bp with a G + C content of 36.5%. The genomic analysis provides significant insights into the antibiotic resistance genes of the genus *Kurthia*.

## ANNOUNCEMENT

The genus *Kurthia* is a gram-positive rod-shaped organism within the *Planococcaceae* family. The first identified species, *Kurthia zopfii,* was isolated from chicken intestines in 1883 (1–3). *Kurthia* species are typically regarded as non-pathogenic, commonly found in the environment or as part of the normal microflora in various animals and plants (3, 4). However, they have been isolated from diverse clinical specimens, including sputum, pilonidal cysts, urethras, aortic valves, and fecal samples from patients with acute diarrhea, suggesting their potential as opportunistic pathogens (4). In this study, we report the draft genome sequence of *Kurthia* strain ISK08, isolated from shrimp in Bangladesh.

The shrimp samples were collected from Dhaka, Bangladesh, following standard procedures. The samples were enriched in Luria-Bertani (LB) broth at 37°C for 18–24 hours. These samples were subsequently plated onto urinary tract infection agar (HiMedia, India) and incubated aerobically at 37°C for 18–24 hours. The *Kurthia* was isolated in tryptic soy agar. Genomic DNA was extracted from a pure colony grown overnight in LB broth using the Qiagen Mini Kit (Qiagen, Germany). DNA purity and concentration were verified using a NanoDrop One Microvolume UV-Vis Spectrophotometer (Thermo Fisher Scientific, USA). The library preparation was performed using the Nextera XT Library Preparation Kit (Illumina, USA). The genome was sequenced on an Illumina MiSeq platform with paired-end sequencing (2 × 150 bp) and 34-fold genome coverage. The number of paired-end reads was 645,651 bp. The quality of the raw sequences was checked using FastQC version 0.11.9 (5). Raw paired-end reads were trimmed using Trimmomatic version 0.39 (6), and genome assembly was performed using Unicycler version 0.4.8 (7). The organism was taxonomically identified using Kraken2 version 2.1.3 (8). The annotation of the genome was carried out using Prokka version 1.14.6 (9). The Rapid Annotations Subsystems Technology (RAST) server was used for structural gene prediction and functional annotation (10). The assembled genome was analyzed for antibiotic resistance genes using ABRIcate version 1.0.1 (11). For the pangenome analysis, Roary version 3.13.0 (12) was utilized with a sequence identity threshold of 95%. The input GFF files required by Roary were generated using Prokka version 1.14.6 (9). Default parameters were used for all software unless otherwise noted.

The draft genome of the *Kurthia* strain ISK08 is 2,885,242 bp long and has an average G + C content of 36.5%. The genome was distributed into 18 contigs with an *N*_50_ and *N*_90_ value of 411,037 and 175,718, respectively. A total of 2,824 coding sequences were identified in the genome, consisting of 1,774 proteins with functional assignments and 1,050 hypothetical proteins. Moreover, 72 RNA genes were detected: 69 transfer RNA, 2 ribosomal RNA genes, and 1 transfer-messenger RNA. Antibiotic-resistant genes for tetracycline, trimethoprim, and lincosamide were detected using the ABRIcate tool. RAST analysis uncovered 261 subsystems with 28% coverage. The draft genome of the *Kurthia* strain ISK08 was compared to 14 complete *Kurthia* genome sequences from the NCBI database for pan-genome analysis. Pan-genomic analysis revealed that the genome of ISK08 is closely related to *Kurthia sp*. 11kri321.

## Data Availability

The *Kurthia* strain ISK08 whole-genome shotgun project has been deposited in DDBJ/ENA/GenBank under accession no. JBJXHL000000000.1. The raw reads were deposited in the NCBI SRA database with accession number SRR31323162.
